# Immuno-Northern Blotting: Detection of RNA Modifications by Using Antibodies against Modified Nucleosides

**DOI:** 10.1371/journal.pone.0143756

**Published:** 2015-11-25

**Authors:** Eikan Mishima, Daisuke Jinno, Yasutoshi Akiyama, Kunihiko Itoh, Shinnosuke Nankumo, Hisato Shima, Koichi Kikuchi, Yoichi Takeuchi, Alaa Elkordy, Takehiro Suzuki, Kuniyasu Niizuma, Sadayoshi Ito, Yoshihisa Tomioka, Takaaki Abe

**Affiliations:** 1 Division of Nephrology, Endocrinology, and Vascular Medicine, Tohoku University Graduate School of Medicine, Sendai, Japan; 2 Tohoku Medical Megabank Organization, Sendai, Japan; 3 Laboratory of Oncology, Pharmacy Practice and Sciences, Tohoku University Graduate School of Pharmaceutical Sciences, Sendai, Japan; 4 Department of Clinical Pharmacology and Genetics, School of Pharmaceutical Sciences, University of Shizuoka, Shizuoka, Japan; 5 Department of Neurosurgery, Tohoku University Graduate School of Medicine, Sendai, Japan; 6 Department of Medical Science, Tohoku University Graduate School of Biomedical Engineering, Sendai, Japan; 7 Department of Clinical Biology and Hormonal Regulation, Tohoku University Graduate School of Medicine, Sendai, Japan; The John Curtin School of Medical Research, AUSTRALIA

## Abstract

The biological roles of RNA modifications are still largely not understood. Thus, developing a method for detecting RNA modifications is important for further clarification. We developed a method for detecting RNA modifications called immuno-northern blotting (INB) analysis and herein introduce its various capabilities. This method involves the separation of RNAs using either polyacrylamide or agarose gel electrophoresis, followed by transfer onto a nylon membrane and subsequent immunoblotting using antibodies against modified nucleosides for the detection of specific modifications. We confirmed that INB with the antibodies for 1-methyladenosine (m^1^A), *N*6-methyladenosine (m^6^A), pseudouridine, and 5-methylcytidine (m^5^C) showed different modifications in a variety of RNAs from various species and organelles. INB with the anti-m^5^C antibody revealed that the antibody cross-reacted with another modification on DNA, suggesting the application of this method for characterization of the antibody for modified nucleosides. Additionally, using INB with the antibody for m^1^A, which is a highly specific modification in eukaryotic tRNA, we detected tRNA-derived fragments known as tiRNAs under the cellular stress response, suggesting the application for tracking target RNA containing specific modifications. INB with the anti-m^6^A antibody confirmed the demethylation of m^6^A by the specific demethylases fat mass and obesity-associated protein (FTO) and ALKBH5, suggesting its application for quantifying target modifications in separated RNAs. Furthermore, INB demonstrated that the knockdown of FTO and ALKBH5 increased the m^6^A modification in small RNAs as well as in mRNA. The INB method has high specificity, sensitivity, and quantitative capability, and it can be employed with conventional experimental apparatus. Therefore, this method would be useful for research on RNA modifications and metabolism.

## Introduction

RNAs contain a wide variety of post-transcriptional nucleoside modifications [1, 2], which vary greatly between different RNA molecules, organisms and organelles. Although their functional importance in human biology has not been extensively studied, it has recently been partially revealed that some modifications modulate a variety of RNA functions and biological processes, and they are linked to various human diseases [3, 4]. For example, pseudouridine is the most abundant modified nucleoside in a wide variety of cellular RNAs containing tRNA, rRNA and small nuclear RNAs (snRNA), and it has been reported to contribute to the RNA-mediated cellular processes [4, 5]. tRNA also contains other various modifications, and tRNA modifications outside the anticodon loop, such as 1-methyladenosine, are generally used to maintain the stability of tRNA or modulate the tRNA folding [4, 6]. In addition, because various types of cell stress dynamically shift the population of tRNA modifications, such stress-related alterations in tRNA are regarded as an adaptive response mechanism, and their detection would therefore be useful for evaluating cell damage [4, 7]. Furthermore, a lack of several tRNA modifications that contribute to proper tRNA function has been associated with various human diseases [4]. For example, alterations in the taurinomethyl modification of mitochondrial tRNA causes mitochondrial diseases [8]. Similarly, an *N*6-methyladenosine modification that is abundant in mRNA as well as in tRNA and rRNA has been reported to be related to cell meiosis and physiological human conditions such as obesity and spermatogenesis [9, 10]. Thus, RNA modifications have been investigated with emphasis on their effects on human health and disease; however, the precise roles of RNA modifications are still largely unknown in many aspects. To further elucidate the biological functions of RNA modifications, the development of analytical methods for detecting RNA modifications and their alterations is needed.

To detect specific modifications or modified bases of RNAs, isolated RNAs are fragmented into nucleosides and then analyzed by physiochemical techniques such as high-performance liquid chromatography or mass-spectrometry (MS). However, the fragmentation step before the analysis may make the original structure of the RNA unclear. Dot blot analysis is also used as an alternative way of detecting specific modifications, and for this method, the fragmentation step is not needed. However, this technique has the limitation that a separation step according to the size of the RNAs is not performed, and hence, it is unable to distinguish which specific RNA is targeted among all of the RNAs in the sample.

Here, we report a protocol for detecting specific RNA modifications named “immuno-northern blot” analysis. The immuno-northern blot is performed using a modified northern blotting procedure with specific antibodies against modified nucleosides. This method does not require fragmentation of the RNAs before analysis, and it also enables the separation of RNAs based on their molecular weights, thus allowing detection of the modifications in different types of RNA. The present method provides a simple and useful technique for analyzing RNA modifications using a conventional experimental apparatus.

## Results

### Immuno-northern blotting using antibodies against modified nucleosides

**Fig 1:**
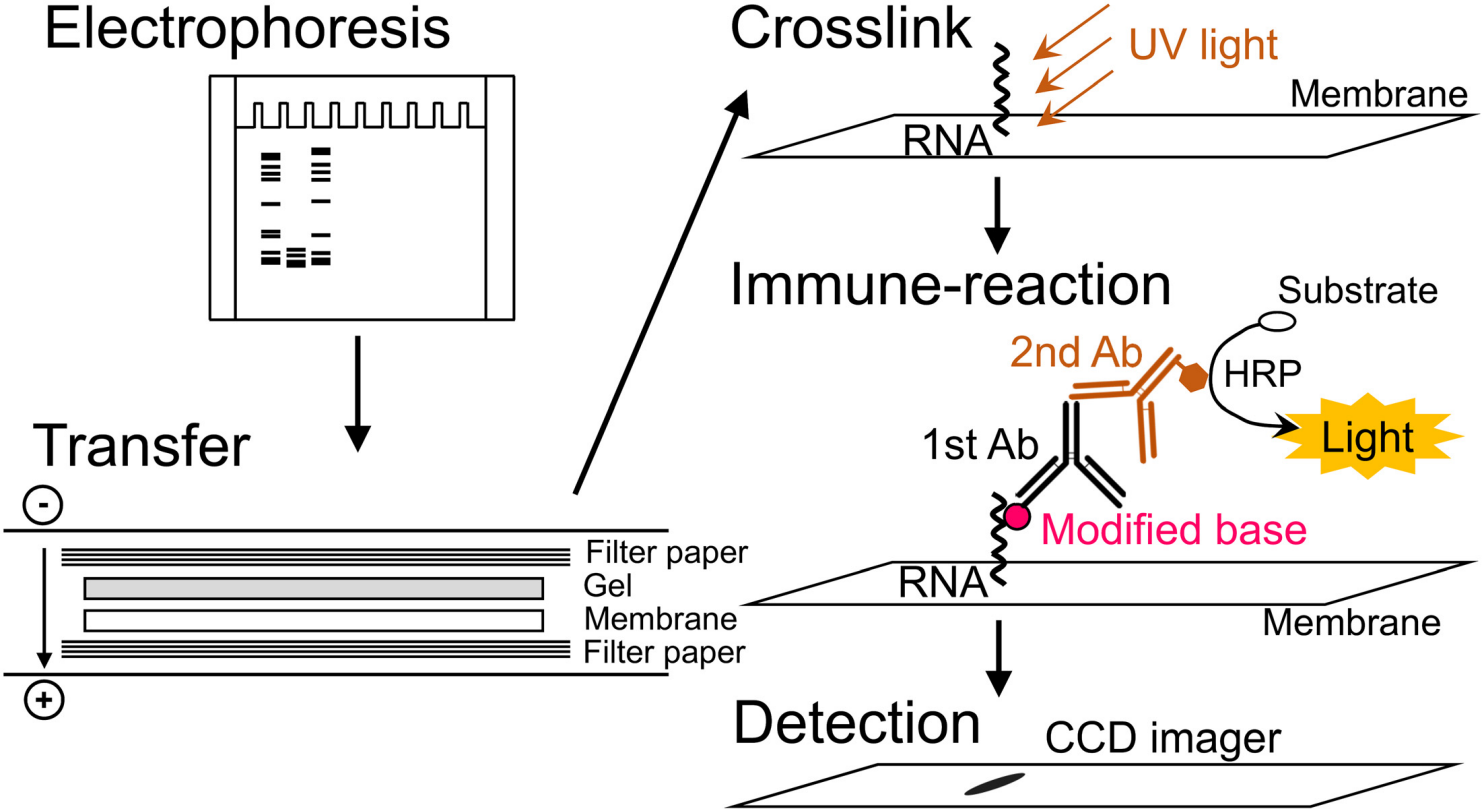
Immuno-northern blotting protocol. In this method, RNAs were separated by denaturing acrylamide or agarose gel electrophoresis, transferred onto a positively charged nylon membrane followed by UV cross-linking, and then incubated with the primary antibodies against the specific modified nucleoside as well as the secondary antibody. The specific bands were visualized by chemiluminescence.

In the present immuno-northern blot analysis (described in detail in Materials and Methods), RNAs are detected by antibodies against the modified nucleosides instead of by the radio-labelled DNA probes used for a conventional northern blot protocol. Briefly, as shown in Fig 1, RNAs are separated in the denaturing acrylamide or agarose gel, transferred onto the nylon membrane, cross-linked by UV irradiation, and then incubated with the primary antibodies against the specific modified nucleoside. The specific bands are detected by subsequent incubation with the secondary antibody and the chemiluminescent reaction. The protocol of this method is similar to that of western blotting.

**Fig 2:**
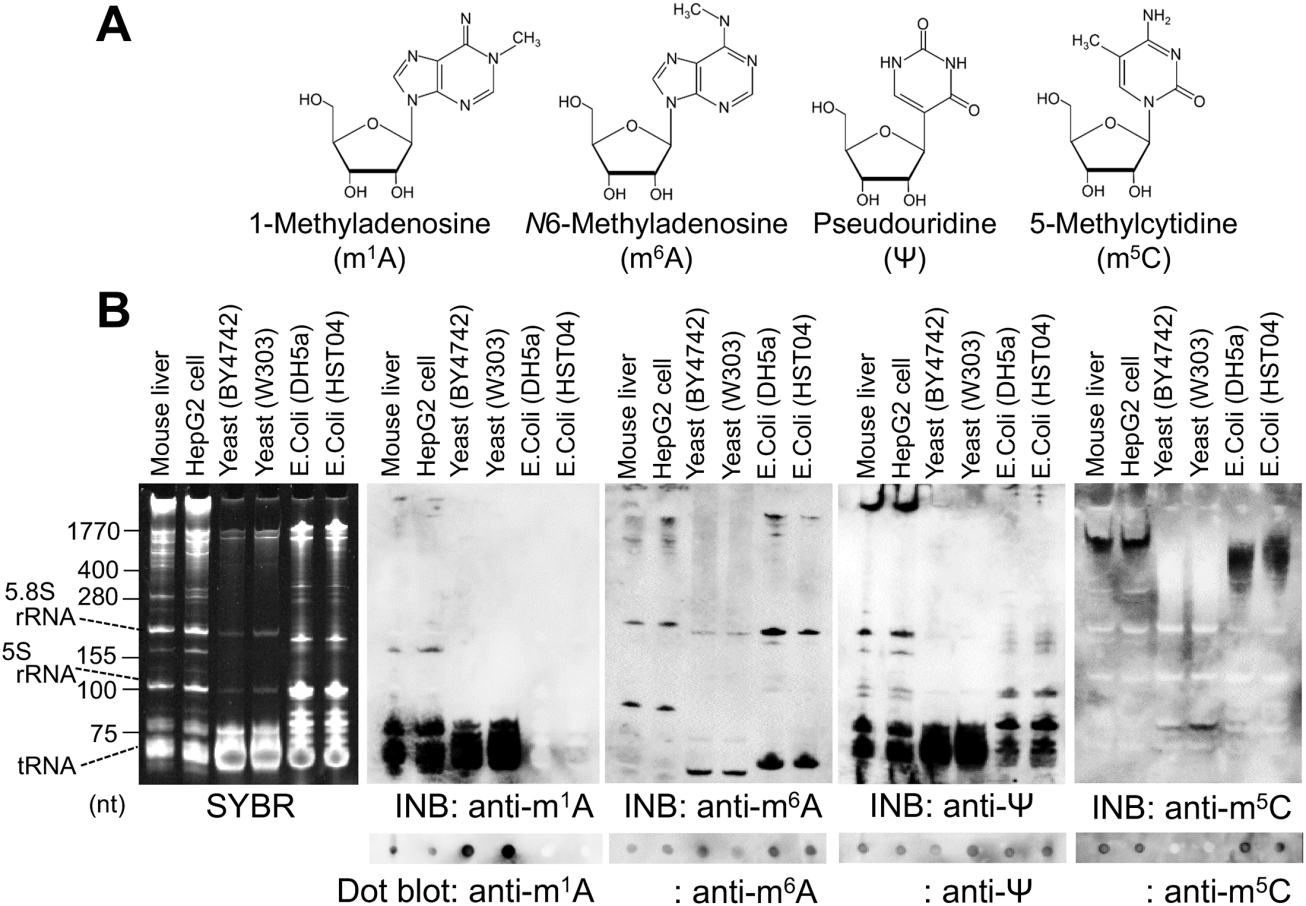
Immuno-northern blotting using antibodies against modified nucleosides. (A) Structural formulas of 1-methyladenosine (m^1^A), *N*6-methyladenosine (m^6^A), pseudouridine (Ψ), and 5-methylcytidine (m^5^C). (B) Total RNA (0.6 μg) isolated from the indicated samples was separated in the 12% polyacrylamide-8 M urea denaturing gel and analyzed by SYBR staining and immuno-northern blotting (INB) using the anti-m^1^A, anti-m^6^A, anti-Ψ and anti-m^5^C antibodies. The total RNA samples (0.6 μg) were also analyzed by the dot blot assay using each antibody, as indicated in the bottom panels.

We performed this immuno-northern blotting (INB) for the detection of the modified nucleosides with the antibodies against 1-methyladenosine (m^1^A), *N*6-methyladenosine (m^6^A), pseudouridine (Ψ), and 5-methylcytidine (m^5^C) in total RNAs isolated from various samples of mammalian cells (mouse liver and human liver HepG2 cell line), yeast (*S*. *cerevisiae* of strain BY4742 and W303), and bacteria (*E*. *coli* of strain DH5α and HST04) (Fig 2A and 2B).

The SYBR staining, a method for staining nucleic acids, detected each of the RNAs, including tRNA, 5S rRNA and 5.8S rRNA, separately and showed the differences in the composition of the RNAs among the mammal, yeast, and bacterial RNAs (Fig 2B). m^1^A is known as a highly conserved modification in eukaryotic RNA and is mainly present in tRNA [11]. Consistent with the previous finding [7], INB by the anti-m^1^A antibody showed the rich m^1^A modification mainly in the tRNA of mammal and yeast RNAs, and no signal was detected in bacterial RNAs. INB by anti-m^6^A antibody showed the positive signal in mammal, yeast and bacterial RNAs, especially in a part of yeast- and bacterial-tRNA (Fig 2B). In mammal RNAs, positive signals detected by the anti-m^6^A antibody were observed in the higher molecular weight RNAs (approximately 1770 nt), the RNAs slightly longer than 5.8S rRNA and slightly shorter than 5S rRNA (Fig 2B). Because the presence of m^6^A modifications was reported in eukaryotic mRNA, 18S rRNA, 28S rRNA, and snRNA [12–14], the positive bands by the anti-m^6^A antibody were probably derived from these m^6^A-containing RNAs. Although both m^1^A and m^6^A are methylated adenosine, these results demonstrated that the antibodies against each methylated-adenosine recognized the difference in the methylated positions of m^1^A and m^6^A, suggesting the high specificity of these antibodies. INB by anti-Ψ antibody ubiquitously showed positive signals in yeast, bacterial, and mammal RNA including tRNA, 5S rRNA and 5.8S rRNA. INB by the anti-m^5^C antibody and acrylamide gel separation showed the positive signal in approximately 1,770 nt of the mammal RNAs, a part of the yeast tRNA (approximately 75 nt), and approximately 1,000 nt of the bacterial tRNA (Fig 2B).

The results of INB using each antibody were consistent with those of dot blot analysis, except for those when using the anti-m^5^C antibody (Fig 2B, bottom panels). Although INB with the m^5^C antibody showed a positive signal in part of yeast tRNA, the dot blot with the antibody did not show a positive signal in the yeast RNA (Fig 2B). Thus, we additionally examined the dot bot with the anti-m^5^C antibody (S1 Fig). As a result, we revealed that the heating pretreatment of the sample affected the result of dot blot with anti-m^5^C antibody. With no pretreatment, the yeast RNA showed a positive signal by the anti-m^5^C dot blot, and mammalian as well as bacterial RNA did not (S1A Fig). In contrast, with heat treatment in the urea-containing sample buffer, the yeast RNA did not show positive signals by the anti-m^5^C dot blot while mammalian and bacterial RNA did (S1B Fig). Because heat denaturing treatment can alter the conformational structures of nucleic acids, we suppose that the conformational condition of the sample affected the binding affinity for anti-m^5^C antibody, thus causing the different results in the INB and dot blot with the anti-m^5^C antibody depending on the treatment conditions of each sample.

### Immuno-northern blotting with agarose gel electrophoresis

**Fig 3:**
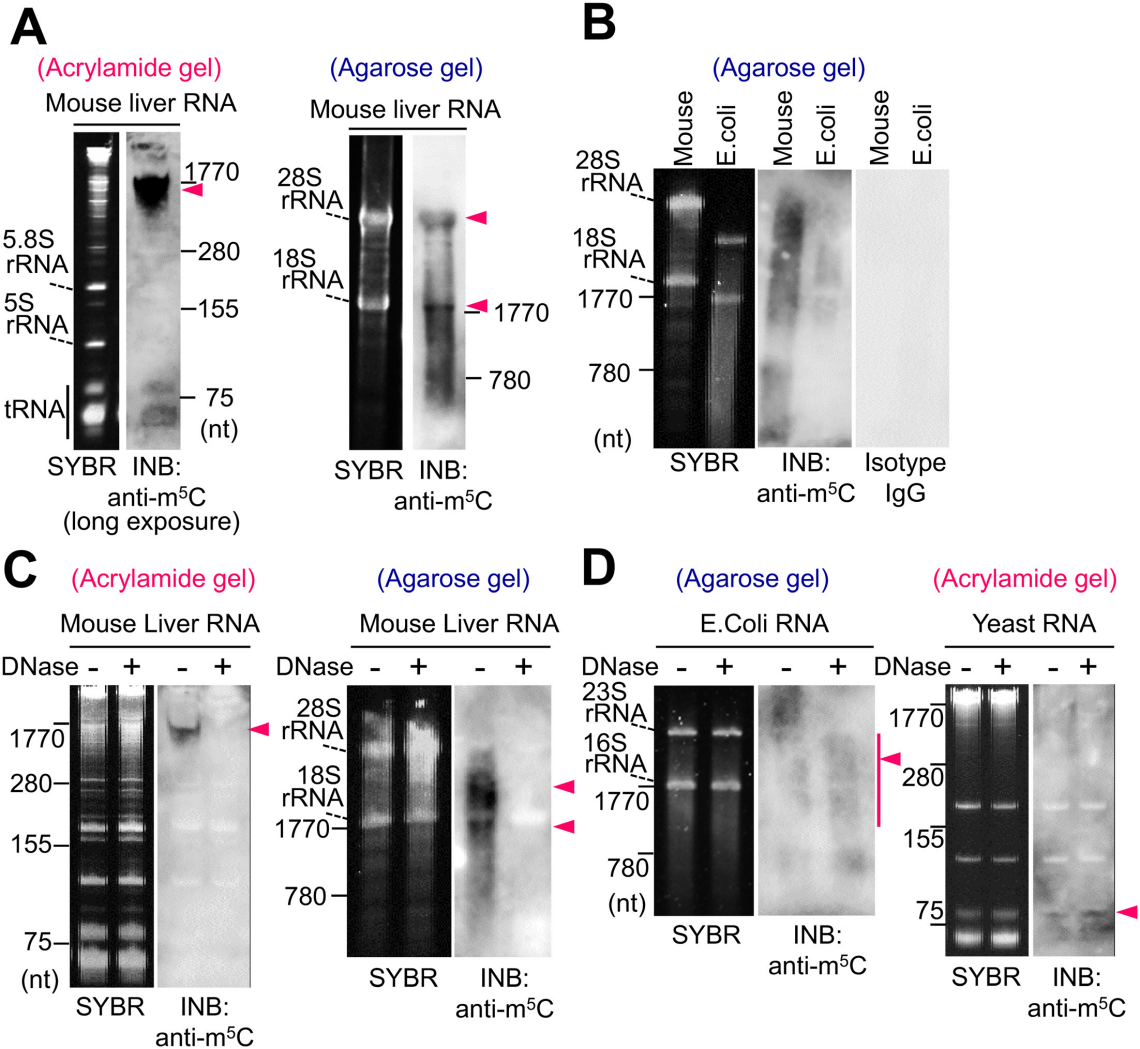
Immuno-northern blot analysis using the anti-m^5^C antibody with acrylamide and agarose gel separation. (A) Total RNA (0.6 μg) isolated from mouse liver was analyzed by SYBR staining and INB with anti-m^5^C antibody after electrophoresis in the 12% polyacrylamide or 1% agarose denaturing gel. The INB image using acrylamide gel is a long-exposure image. Arrowheads denote the positive signals, which appear to be integrated in the INB image using the acrylamide gel. (B) Total RNA isolated from mouse liver (1.2 μg) and *E*. *coli* HST04 (0.6 μg) was analyzed by SYBR staining and INB with anti-m^5^C antibody or isotype IgG. As a negative control for INB, isotype IgG was used instead of anti-m^5^C antibody at the same concentration. (C, D) The effect of DNase I treatment on the anti-m^5^C positive signal. Total RNAs isolated from mouse liver (1.2 μg, C), *E*. *coli* HST04 (0.6 μg, D), and yeast BY4742 (1.0 μg, D) were treated with or without DNase I, and then it was analyzed by SYBR staining and INB with anti-m^5^C antibody using the indicated gel. Arrowheads denote the positive signals.

Acrylamide gel electrophoresis is optimal for the analysis of small RNAs because of its high resolution capability of RNAs as shown in Fig 2B, but it is unsuitable for the analysis of high molecular weight RNAs. Thus, next we performed INB using agarose gel electrophoresis for the separation of the RNA to analyze high molecular weight RNAs. Agarose gel separated the high molecular weight RNAs and distinguished between 28S and 18S rRNA (Fig 3A). Although a positive signal was detected at approximately 1,770 nt as an integrated band (Figs 2B and 3A) in the INB by anti-m^5^C antibody using acrylamide gel separation, the agarose gel based-INB separated the anti-m^5^C positive signals including the signals corresponding to around 18S and 28S rRNA (Fig 3A).

However, currently the presence of the m^5^C modification has not been reported in eukaryotic 18S rRNA [15]. Therefore, to exclude the possibility that the signals were non-specific signals by the anti-m^5^C antibody, we performed INB using mouse isotype IgG as a negative control instead of the anti-m^5^C antibody. Because isotype IgG did not show any positive bands in the mammal and bacterial RNAs (Fig 3B), the positive signals in the m^5^C antibody based-INB were unlikely to be non-specific signals by the antibody. Next, we examined the effect of contaminating DNA in isolated RNA samples on the anti-m^5^C positive signal because the anti-m^5^C antibody used in the present study (clone FMC-9) was reported to have a strong cross-reactivity with 5-methyl-2'-deoxycytidine that is present in DNA [16]. To eliminate the effect of contaminating DNA, we treated the isolated RNA with DNase I (Fig 3C and 3D). As a result, DNase I treatment diminished the m^5^C-positive signal including around 18S and 28S rRNA in the mammalian RNA (Fig 3C). These data suggest that the anti-m^5^C positive signals in mammalian RNAs were not derived from RNAs but from the 5-methyl-2'-deoxycytidine on the contaminating DNA component in the RNA sample. In contrast, DNase I treatment did not diminish the m^5^C-positive signal in the bacterial and yeast RNAs (Fig 3D), suggesting that the anti-m^5^C positive signals in the bacterial and yeast RNAs showed the m^5^C modification in the RNA components.

### Analysis of intracellular localization of RNA modifications

**Fig 4:**
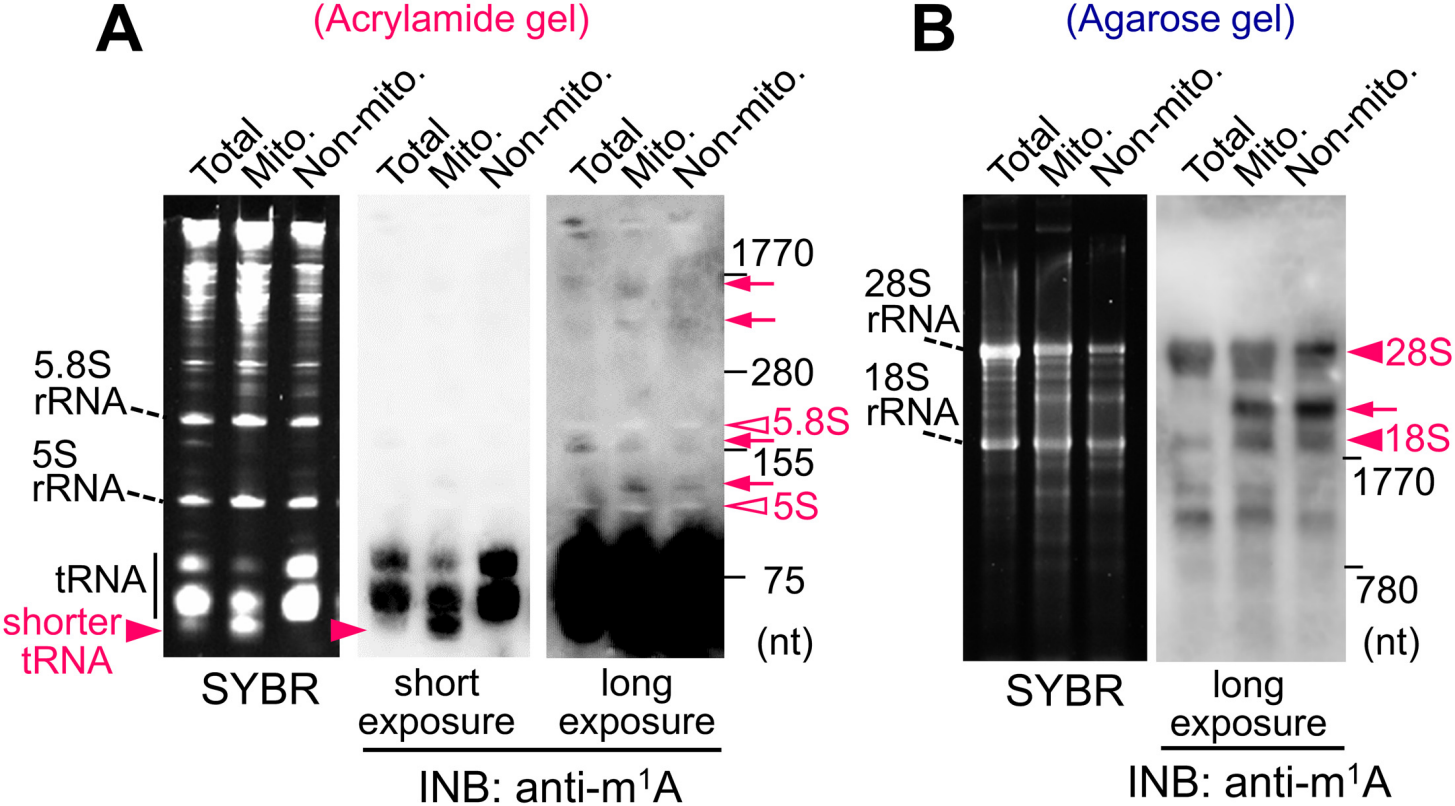
Immuno-northern blot analysis of mitochondrial and non-mitochondrial RNA. RNAs were isolated from the total fraction (Total), mitochondrial fraction (Mito.), and non-mitochondrial fraction (Non-mito.) of mouse liver. (A) Each total RNA (0.6 μg) was separated in the 12% polyacrylamide gel and analyzed by SYBR staining and INB with anti-m^1^A antibody. The red arrowheads denote shorter tRNA bands, which were rich in the mitochondrial RNA. Arrows denote slight anti-m^1^A positive bands in the long exposure image. White arrowheads denote negative signals in 5.8S and 5S rRNA. (B) Each total RNA (1.2 μg) was separated on the 1% agarose gel and analyzed. The arrowheads denote positive signals in 28S and 18S rRNA. The arrow probably denotes the 28S rRNA-derived degraded transcript produced during the sample preparation.

Mitochondria have different tRNA compositions and modification patterns compared to those in cytoplasm [17, 18]. The m^1^A modification is known to be present in eukaryotic mitochondrial tRNA as well as in cytoplasmic tRNA [18]. Using INB, we next examined the intracellular localization of the m^1^A modification in the mitochondria and cytoplasm. RNAs isolated from the total, mitochondrial, and non-mitochondrial fraction of mouse liver were separated in an acrylamide gel and then analyzed by SYBR staining and INB (Fig 4A). The SYBR staining showed that the smaller-sized tRNA of 70 nt was present to a greater extent in the mitochondrial fraction than in the non-mitochondrial fraction (Fig 4A, arrowhead). The INB by anti-m^1^A antibody showed that both mitochondrial and non-mitochondrial tRNA contain the m^1^A modification (Fig 4A). In addition to tRNA, 18S and 28S rRNA among eukaryotic RNAs have been reported to contain m^1^A modifications, but 5S and 5.8S rRNA have not [1]. Consistent with these previous findings, the INB with agarose gel separation and anti-m^1^A antibody showed positive bands in 28S and 18S rRNA, indicating the presence of m^1^A in these rRNAs (Fig 4B). Additionally, the INB with acrylamide gel separation showed negative signals at approximately 120 and 160 nt that were identical positions to 5.8S and 5S rRNA in SYBR staining, respectively (Fig 4A, white arrowheads), suggesting the lack of the m^1^A modification in 5S and 5.8S rRNA. In the long exposed image of INB by anti-m^1^A, slight m^1^A positive-bands were detected in RNAs other than tRNA both in the mitochondrial and non-mitochondrial fractions (Fig 4A, arrows). These data indicate the presence of minor m^1^A modifications in other RNA species as well as in tRNA.

### tRNA-derived fragments detected by immuno-northern blotting

**Fig 5:**
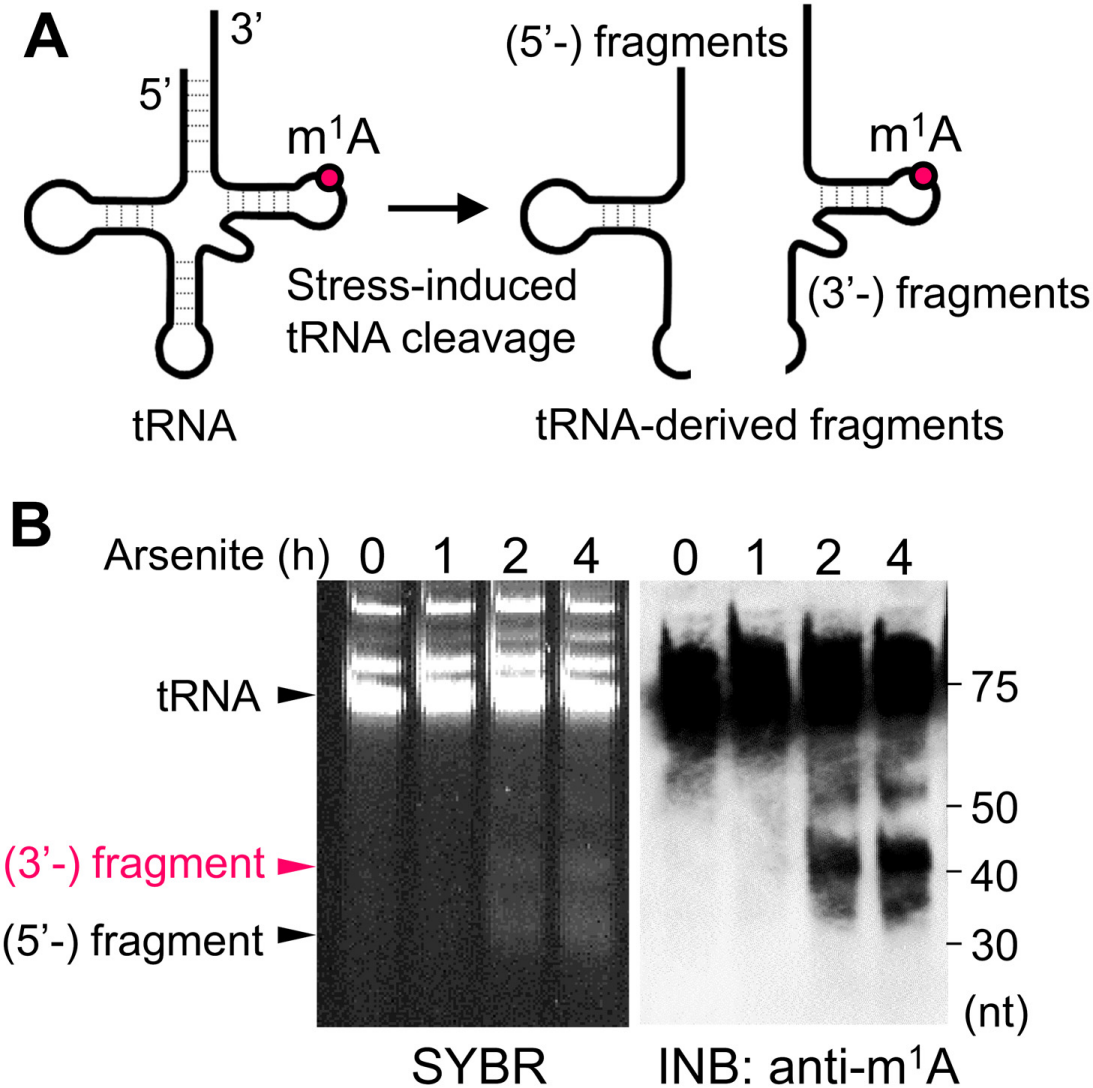
Detection of stress-induced tRNA-derived fragments by immuno-northern blotting. (A) The image of stress-induced tRNA cleavage. tRNAs are cleaved under conditions of cell stress, and consequently, tRNA-derived fragments such as the 5’-side and 3’-side fragments appear. The modification of m^1^A is usually contained in the 3’ side of the tRNA structure. (B) Total RNA (0.75 μg) was isolated from HK-2 cells treated with sodium arsenite (500 μM) for the indicated periods and analyzed by SYBR staining and INB with anti-m^1^A antibody using acrylamide gel separation. Arsenite-stress induced tRNA cleavage, and the resultant tRNA-derived fragments were detected by SYBR staining and firmly detected by the INB with anti-m^1^A antibody.

Various cell stresses, such as oxidative stress, induce intracellular tRNA cleavage resulting in tRNA-derived stress-induced fragments known as tiRNAs or tRF (Fig 5A) [7, 19]. Using INB, we examined the stress-induced tRNA cleavage in human kidney HK-2 cells. SYBR staining showed that arsenite, an inducer of oxidative stress, induced shorter bands of approximately 40 nt than those of full length tRNA (Fig 5B). The INB with the anti-m^1^A antibody clearly detected short, positive bands in addition to full length tRNA in the arsenite-treated cells (Fig 5B). These results demonstrated that the short bands were stress-induced tRNA-derived fragments because the m^1^A modification is highly specific in eukaryotic tRNA at position 58 in the 3’-half side (Figs 2B and 5A). These results suggest that INB is also suitable for tracking the changes in specific RNAs containing target modifications, such as the m^1^A modification in tRNA-derived fragments, as previously reported [7].

### Specificity, sensitivity, and quantitative capability of immuno-northern blotting

**Fig 6:**
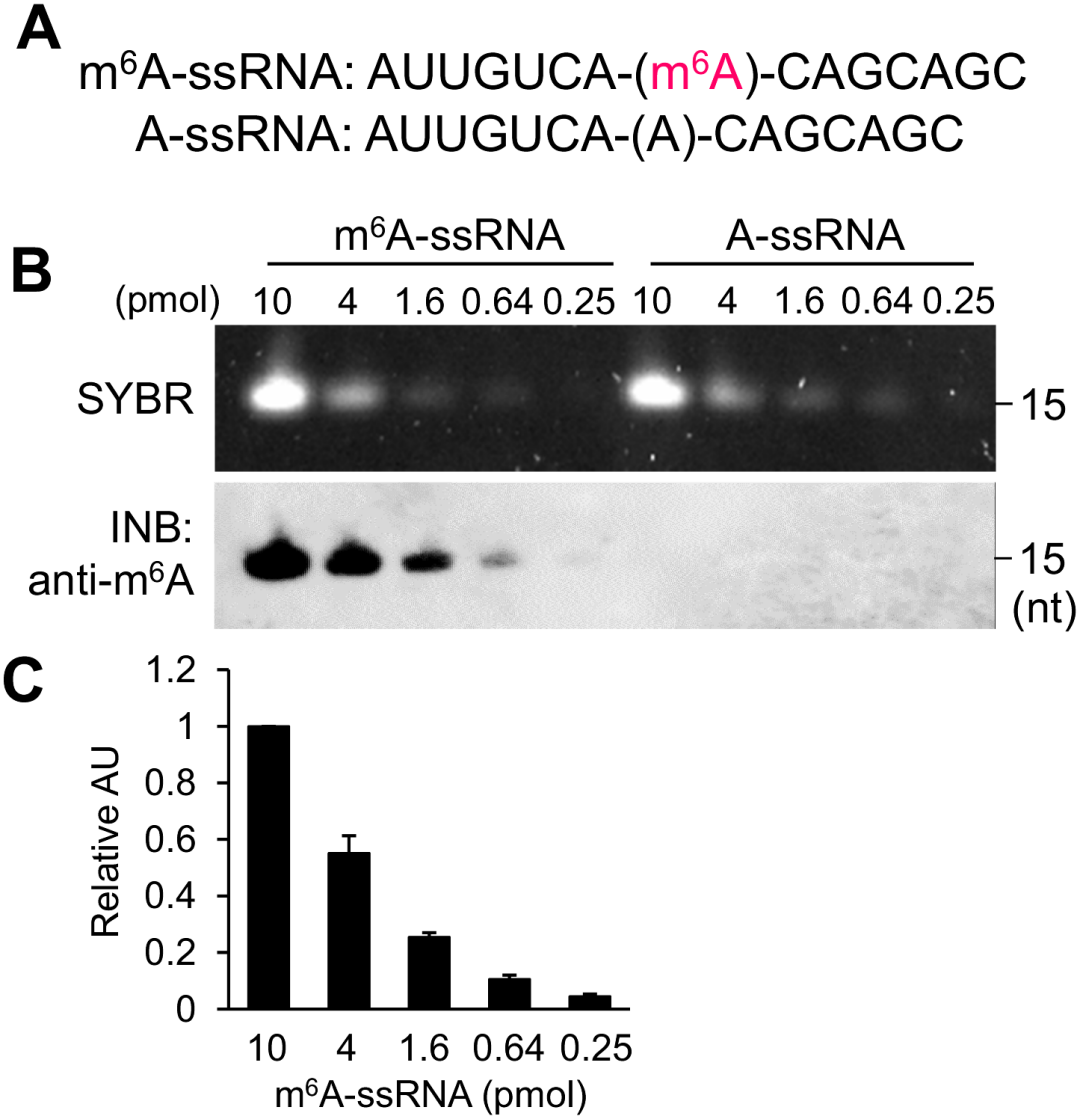
Specificity and sensitivity of immuno-northern blotting. (A) The sequences of synthesized 15-mer ssRNA containing either m^6^A or A at the center position. (B) SYBR staining and INB with anti-m^6^A antibody. Serially diluted m^6^A- and A-containing 15-mer ssRNA (10, 4, 1.6, 0.64, and 0.25 pmol) were analyzed. (C) Relative band intensities by INB of m^6^A-containing ssRNA. Densitometric analysis was performed to quantitate the band intensities. Data were expressed relative to the band intensity of each 10 pmol sample, which was taken as 1.0 arbitrary units (AU), N = 3. Values are shown as the mean ± SD.

Next, we evaluated the specificity, sensitivity, and quantitative capability of INB. We synthesized 15-mer single strand RNA (ssRNA) with a site-specific incorporated m^6^A base (Fig 6A), and it was analyzed using SYBR staining and INB with anti-m^6^A antibody. Both SYBR staining and INB detected an amount of ssRNA at the 0.64 pmol level, and furthermore, INB was able to detect a concentration as low as 0.25 pmol of m^6^A-containing ssRNA (Fig 6B). INB with the anti-m^6^A antibody did not show any bands in the ssRNA samples without the m^6^A modification (Fig 6B), indicating the high specificity of the INB analysis and the antibody. Furthermore, the band-intensity of INB was proportional to the amount of applied m^6^A-containing RNAs (Fig 6C), suggesting the quantitative capability of the analysis.

### Evaluation of m^6^A-demethylation by INB

**Fig 7:**
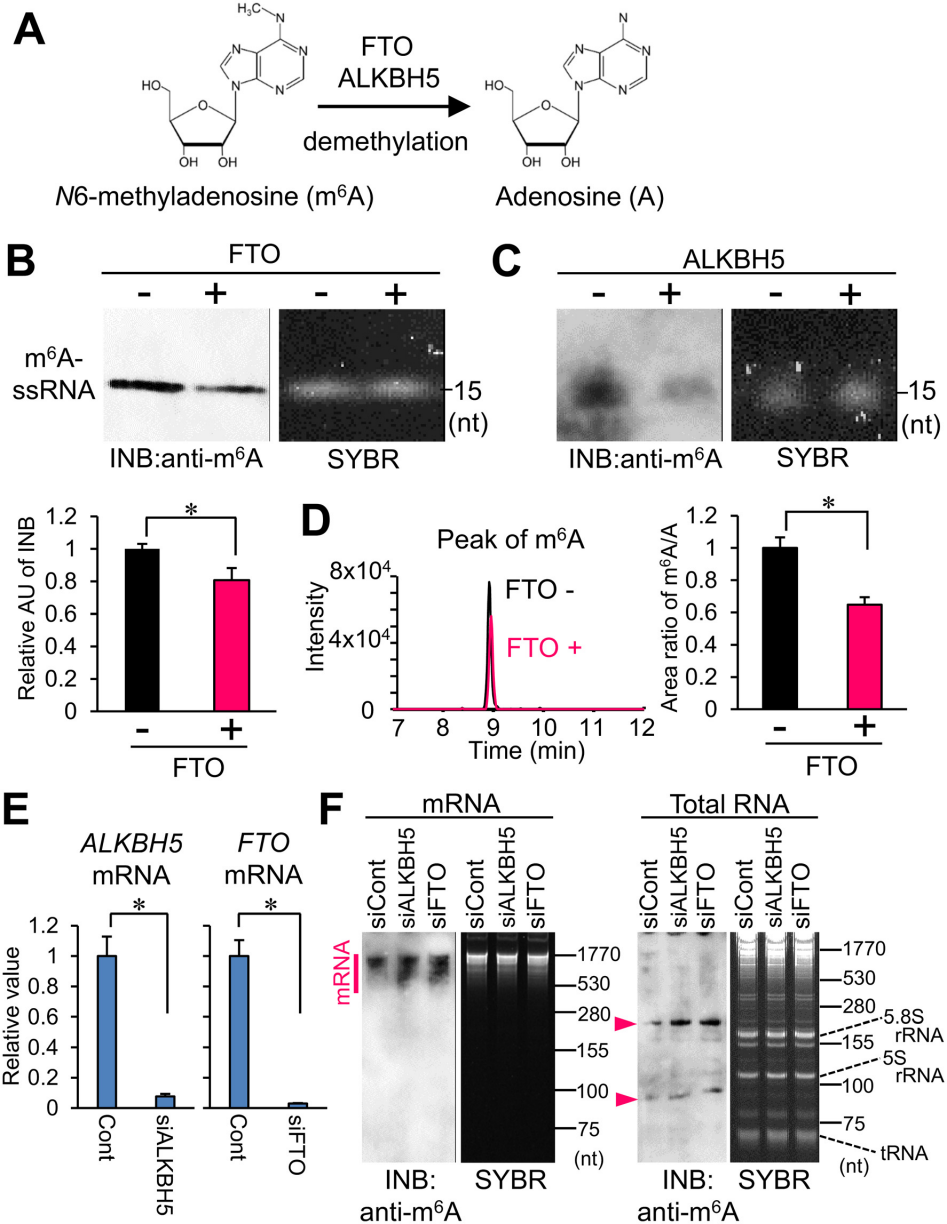
Detection of m^6^A-demethylation by immuno-northern blotting. (A) Demethylation of m^6^A to A in RNA by FTO and ALKBH5. (B) m^6^A-containig 15 mer-ssRNA was treated with or without FTO and analyzed by INB with anti-m^6^A antibody and SYBR. The bottom graph shows a densitometric quantification of the band intensity. Data were expressed relative to the mean value of the FTO (-) samples, which was taken as 1.0 AU, N = 3. (C) m^6^A-containig 15 mer-ssRNA was treated with or without ALKBH5 and analyzed by INB and SYBR. A representative image is shown. (D) LC-MS/MS analysis of FTO-mediated demethylation. After the FTO treatment, m^6^A-ssRNA was digested and subsequently analyzed by LC-MS/MS. Chromatograms of the m^6^A peak of digested nucleosides are shown. The right graph shows a quantification of the relative area ratio of m^6^A/A. Data were expressed relative to the mean value of the FTO (-) samples, which was taken as 1.0. (E) Knockdown of FTO and ALKBH5 in HeLa cells. The knockdown efficiency was measured by quantitative PCR. Relative abundance of each transcript was normalized by *GAPDH*. Data were expressed relative to the mean value of the control, which was taken as 1.0, N = 3. (F) Evaluation of the m^6^A modification in RNAs in FTO- and ALKBH5-knockdown HeLa cells. The mRNA (100 ng) and total RNA (1.0 μg) were analyzed by INB with anti-m^6^A antibody using acrylamide gel separation. Arrowheads denote the anti-m^6^A positive signals. SYBR staining is shown for the loading control. Values are shown as the mean ± SD. *P < 0.05.

Fat mass and obesity-associated protein (FTO) and AlkB family member 5 (ALKBH5) have been shown to have demethylation activity targeting m^6^A residues in RNAs (Fig 7A) [9, 10]. We evaluated the *in vitro* demethylation of m^6^A by FTO and ALKBH5 using INB. After the treatment with or without recombinant human FTO and ALKBH5, m^6^A containing-ssRNA was analyzed with SYBR staining and INB using anti-m^6^A antibody (Fig 7B and 7C). Although SYBR staining showed no differences in the amounts of RNA between the samples, INB revealed demethylation at 20% of the total m^6^A in the m^6^A containing-ssRNA by treatment with FTO (Fig 7B). The demethylation of m^6^A by treatment with recombinant ALKBH5 was also shown by the INB with anti-m^6^A antibody (Fig 7C). To confirm the quantification of the m^6^A-demethylation by FTO, we measured the m^6^A residues in the sample by using liquid chromatography-MS/MS (LC-MS/MS). Assessment of m^6^A-demethylation by LC-MS/MS showed results similar to those by INB (Fig 7D). These results suggest that INB is suitable for evaluating the quantitative variation of a specific modification in the target RNA.

Furthermore, we examined whether INB can evaluate the endogenous m^6^A-demethylation inside cells. In human HeLa cells, ALKBH5 and FTO were knocked down by siRNA. The siRNA of ALKBH5 and FTO decreased the level of each mRNA to 7.1% and 3.0%, respectively (Fig 7E). Total RNA and mRNA were isolated from HeLa cells following the knockdown of FTO or ALKBH5. We evaluated the relative level of the m^6^A modification in the total RNA and mRNA by INB with anti-m^6^A antibody (Fig 7F). As a result, INB analysis showed an increase in the m^6^A modification in mRNA by the knockdown of ALKBH5 and FTO (Fig 7F). In addition, INB using total RNA revealed that the m^6^A modification in small RNAs (slightly longer than 5.8S rRNA and slightly shorter than 5S rRNA) as well as in the mRNA also increased by the knockdown of ALKBH5 and FTO (Fig 7F). These data suggest that INB also can be used to evaluate alterations in the levels of m^6^A-methylation in various RNAs inside cells.

## Discussion

Herein we report INB analysis using antibodies against modified nucleosides and an RNA separation technique by gel electrophoresis. Using this method, we could detect and quantify the target modifications in various separated RNAs. In the present study, INB could reveal the character of the m^5^C antibody that cross-reacts with another DNA modification. Additionally, INB could trace changes in the size of specific RNA, such as stress-induced tRNA cleavage. Furthermore, the results of INB suggest that the m^6^A modification in small RNAs as well as in mRNA are also demethylation substrates for FTO and ALKBH5. Thus, INB analysis provides a simple and useful method for examining RNA modifications without using complicated devices.

The INB analysis does not require the RNA fragmentation that is needed before analysis by the conventional method using high-performance liquid chromatography or MS. RNAs are present not only in their full-length form but also in derivative forms of different length such as tRNA-derived stress-induced fragments (Fig 5). Hence, fragmentation may obscure the original structure of the RNA as well as the whereabouts of the target modification. In contrast, INB without the fragmentation can examine native lengths of RNAs and show which length of the RNA contains the specific modification.

Because transcriptome-wide mapping studies have recently identified the m^6^A modification sites in mRNA, the biological functions of the m^6^A modification are highlighted [14, 20]. In the present study, the results of INB showed an increase in the endogenous m^6^A methylation in mRNA by the knockdown of FTO and ALKBH5 (Fig 7F), which is consistent with previous reports [9, 10]. Additionally, the results of INB suggest that the m^6^A modification in small RNAs as well as in mRNA might be demethylation substrates by FTO and ALKBH5 (Fig 7F). The m^6^A-methylated small RNAs that are potentially targeted by FTO and ALKBH5 were slightly longer than 5.8S rRNA and slightly shorter than 5S rRNA (Fig 7F). Because previous reports have shown that among snRNAs U2, U4, and U6 RNA contain a m^6^A base [13], we suppose that the m^6^A-methylated small RNAs in the present study are such snRNAs or their derivatives. However, to identify the specific RNA composed of the anti-m^6^A positive bands, we need to develop further approaches such as isolating the RNA that constitutes the specific bands by gel excision after separation and using a combination of sequencing methods. Because we have not yet developed such a technique, it will be our challenge in the next study.

INB revealed the character of the antibodies for RNA modification in the present study. The present INB method requires an antibody against a specific modification. For establishing the antibody against the modified nucleoside [16, 21, 22], we prepared an immunogen containing the targeted modified nucleosides by conjugation to a carrier protein [23] due to the low antigenicity of modified nucleosides alone. Although the antibodies for m^1^A, m^6^A and Ψ have high specificities to the target modifications (Fig 6B)[21, 22], the anti-m^5^C antibody used in the present study (clone FMC-9) has been reported to have a 10-fold higher affinity for 5-methyl-2’-deoxycytidine, a modification of DNA, than for m^5^C [16]. Such cross-reactivity of the antibody may lead to some ambiguities of the results. In the present study, the INB revealed that the positive signals of mammalian RNA in the INB with the anti-m^5^C antibody were not derived from the RNA component but from contamination with DNA components (Fig 3C). Using INB, we detected the presence of m^5^C in the RNA of yeast and bacteria; however, we did not detect it in the mammalian RNA (Fig 3C and 3D), possibly because of the low sensitivity of the anti-m^5^C antibody for m^5^C. Additionally, the results of INB and dot blot using the anti-m^5^C antibody were different depending upon the manner of treatment of the samples (Fig 2B and S1 Fig). For example, yeast RNA showed a positive signal by the INB with the anti-m^5^C antibody and by dot blot with no denaturing pretreatment; however, dot blot with the anti-m^5^C antibody and heat denaturing pretreatment in urea-containing buffer did not show positive signals (Fig 2B and S1 Fig). Because heat denaturing treatment can alter the conformational structure of RNA, we suppose that the conformational condition of the sample affected the binding affinity for the anti-m^5^C antibody, resulting in the different signals in the INB and dot blot depending on the treatment conditions for each sample. However, because we have not yet discovered the precise mechanism pertaining to this issue, we are going to address this issue in a future study.

We used a positively charged nylon membrane Hybond N+ (GE Healthcare) as a transfer membrane, which is optimized for the conventional northern blotting protocol. It should be noted that some nylon membranes from different manufacturers are not suitable for the INB protocol, although we do not know the reason for the differences among the products. In the present study, the demethylation efficiency by recombinant FTO was modest (Fig 7B), although the previous report showed a higher demethylation efficiency by FTO than that found in our study [10]. We suppose that the difference between the present result and the previous result was caused by differences in the character and enzymatic activity of the recombinant FTO protein.

In summary, we developed an INB method using antibodies against modified nucleosides. This method is simple and useful for investigating the modifications and size changes of RNAs, and it should be useful for researching nucleic acid modifications and metabolism.

## Materials and Methods

### Materials

Mouse monoclonal anti-1-methyladenosine (clone AMA-2, IgG2b kappa subclass), anti-pseudouridine (clone APU-6, IgG1 kappa subclass), and anti-5-methylcytidine antibody (clone FMC-9, IgG2a lambda subclass) were established as described previously [7, 16, 21, 22] and available for purchase from BML life science. Other antibodies and materials were purchased as follows: rabbit polyclonal anti-*N*6-methyladenosine antibody (Synaptic Systems), horseradish peroxidase (HRP)-conjugated goat anti-mouse IgG (Thermo), HRP-conjugated goat anti-rabbit IgG (Thermo), mouse IgG2a lambda (Sigma-Aldrich), DNase I (RNase-free, Qiagen), RNase inhibitor (Roche), sodium meta-arsenite (Wako), recombinant human FTO protein (Abcam), and recombinant human ALKBH5 protein (Abnova). The m^6^A-incorporated 15-mer ssRNA was synthesized and purified by HPLC (Gene Design) according to the sequence in the literature [10]. Human hepatocarcinoma HepG2 cells (HB-8065), human kidney HK-2 cells (CRL-2190), and human cervical carcinoma HeLa cells (CCL-2) were purchased from ATCC. *E*. *coli* (strain DH5α and HST04) was purchased from Takara-Bio. *S*. *cerevisiae* (strain BY4741 and W303-1B) was a kind gift from Prof. Shusuke Kuge (Tohoku Pharmaceutical University).

### RNA isolation

The total RNAs were isolated from C57BL/6 male mouse liver, human hepatocarcinoma HepG2 cells, *S*. *cerevisiae* (strain BY4741 and W303-1B), and *E*. *coli* (strain DH5α and HST04) using Tripure isolation reagent (Roche) and miRNeasy Mini kit (Qiagen). The mitochondria and non-mitochondrial fractions were isolated from mouse liver as previously described [24, 25]. The RNA concentration was measured by a Bio-Spec Nano spectrophotometer (Shimadzu). To eliminate the contaminating DNA in the isolated RNA solution, we performed optional on-column DNase I digestion with standard RNA isolation using miRNeasy. All animal experiments were approved by the Animal Committee of Tohoku University School of Medicine. Mice were euthanized by cervical dislocation, and tissues were immediately dissected and snap-frozen in liquid nitrogen. A total 6 mice were examined in the present study.

### Immuno-northern blot analysis

An isolated RNA solution with an equal volume of sample buffer (90 mM Tris-borate, 2 mM EDTA, pH 8.3, 8 M urea, 10% sucrose, 0.05% bromophenol blue, and 0.05% xylene cyanol) was denatured by heating at 65°C for 15 min, followed by chilling on ice. Denatured samples were separated by 12% polyacrylamide-8 M urea denaturing gel electrophoresis at 250 V in 0.5x TBE buffer (45 mM Tris, 45 mM borate, and 1 mM EDTA, pH = 8.0). For blotting, separated RNA was transferred onto Hybond N+ positive charged nylon membranes (GE Healthcare) by semidry electroblotting in 0.5x TBE buffer at 300 mA for 60 min. After UV crosslinking at 120,000 μJ/cm^2^ in a Stratalinker UV crosslinker (Stratagene), the membrane was blocked with 2% Block-Ace (Dainihon Pharmaceutical) in TBS-T (50 mM Tris, 150 mM sodium chloride, and 0.1% Tween 20, pH 7.4) for 1 h at room temperature and then incubated with the primary antibodies (diluted 1:500 for anti-m^1^A, anti-PU and anti-m^5^C and 1:1000 for anti-m^6^A) in Can Get Signal solution 1 (Toyobo) overnight at 4°C or for 2 h at room temperature (for anti-m^5^C). After washing with TBS-T, membranes were incubated with appropriate HRP-conjugated secondary antibody diluted 1:1000 in Can Get Signal solution 2 (Toyobo) for 1 h at room temperature. The membrane was visualized by Pierce Western Blotting Substrate Plus (Thermo) and a LAS-4000 mini luminescent image analyzer (Fuji Film). The quantification of the band intensity was analyzed using Multi-Gauge v3.2 software (Fuji Film).

### Immuno-northern blot analysis using agarose gel

The RNA sample was denatured by heating at 65°C for 15 min in 50% formamide and 4.4 M formaldehyde, and then, it was electrophoresed in 1% agarose gels containing 2.2 M formaldehyde in 1x MOPS buffer (pH 7.0). For blotting, separated RNA was transferred onto Hybond N+ nylon membranes by upload capillary blotting with 10x SSC buffer overnight. The following procedures were performed in the same manner as the INB protocol using polyacrylamide gels, which was described above.

### Nucleic acid staining and dot-blotting

For nucleic acid staining, the electrophoresed gel was stained with SYBR Gold Nucleic Acid Gel Stain (Invitrogen). For dot-blot analysis, RNA solution (2.0 μL) was spotted onto the nylon membrane and then treated in the same manner as the INB protocol. For the dot-blot analysis with the anti-m^5^C antibody, a RNA solution with an equal volume of the urea-containing sample buffer was denatured by heating at 65°C for 15 min, followed by chilling on ice, and then, it was spotted on the membrane.

### Cell culture

HepG2 cells were incubated in RPMI1640 supplemented with 10% fetal calf serum. HK-2 and HeLa cells were incubated in DMEM supplemented with 10% fetal bovine serum. Cells were cultured at 37°C in a humidified atmosphere of 95% air-5% CO_2_. For the cell stress assay, HK-2 cells were incubated with the culture medium containing 500 μM sodium arsenite for 1, 2 or 4 hours. After the arsenite exposure, the medium was removed, and then, the total RNA was extracted from the arsenite-treated cells.

### Demethylation assay

The demethylation assay with FTO and ALKBH5 was performed as reported in the literature [9, 10]. For the assay with FTO, a 100 μL aliquot of reaction mixture containing 100 pmol m^6^A-ssRNA with 25 pmol of recombinant FTO, 283 μM (NH_4_)_2_Fe(SO_4_) _2_·6H2O, 300 μM α-ketoglutaric acid, 2 mM L-ascorbic acid, 50 μg/mL bovine serum albumin, and 50 mM HEPES buffer (pH 7.0) was incubated for 3 h at room temperature. For the assay with ALKBH5, a 130 μL aliquot of reaction mixture containing 40 pmol m^6^A-ssRNA with 12 pmol of recombinant ALKBH5, 150 μM (NH_4_)_2_Fe(SO_4_) _2_·6H2O, 100 mM KCl, 2 mM MgCl2, 300 μM α-ketoglutaric acid, 2 mM L-ascorbic acid, 1 U/μL RNase inhibitor, and 50 mM HEPES buffer (pH 7.0) was incubated for 3 h at room temperature. After the incubation with FTO or ALKBH5, the reaction mixture was deproteinized by the Tripure-chloroform procedure and then desalted using Illustra MicroSpin G-25 columns (GE Healthcare). The extracted RNA solution was analyzed by immuno-northern blotting.

### Quantitative analysis of the m^6^A level using LC-MS/MS

The m^6^A and A levels were measured by LC-MS/MS. The sample preparation procedures of m^6^A and A were performed as described in a previous study [7, 26, 27].

LC-MS/MS was comprised of a NANOSPACE SI-2 HPLC system and a TSQ Quantum Ultra (Thermo Fisher Scientific) triple quadrupole mass spectrometer equipped with a heated electrospray ionization source. Samples were analyzed in the selected reaction monitoring mode. The optimum value of the selected reaction monitoring was determined by monitoring the following transitions: *m/z* 282.1 > 150.1 for m^6^A and *m/z* 268.1 > 136.1 for A. Liquid chromatographic separation was performed in the reversed-phase mode with a gradient elution.

### Knockdown of FTO and ALKBH5

Transfections were performed using Lipofectamine RNAiMAX (Invitrogen) following the manufacturer’s protocols. For the knockdown of FTO and ALKBH5 expression, Silencer Select Pre-designed siRNA for FTO and ALKBH5 (Thermo Fisher, siFTO: s29688, s29687, s29688, and siALKBH5: s35510, s35511, s35512) were used. The scrambled RNAi oligo was used as a negative control. Forty-eight hours after the transfection of siRNA into HeLa cells, the total RNA was isolated from the cells with DNase I treatment. mRNA was isolated from the total RNA using a Dynabeads mRNA Purification Kit (Ambion). cDNA synthesis and quantitative PCR analysis were performed as described previously [28]. Gene specific primers and probes were purchased from Applied Biosystems (FTO: Hs01057145_m1; and ALKBH5: Hs00539502_m1).

### Statistics

Values are presented as the mean ± SD. Statistical analysis was evaluated by the unpaired *t* test. Values of *P* < 0.05 were considered to be statistically significant.

## Supporting Information

S1 Fig Dot blot assay with anti-m^5^C antibody. The total RNA samples (0.6 μg) were analyzed by dot blot with anti-m^5^C antibody. (A) Isolated RNAs eluted in water were directly spotted on the membrane. Arrowheads indicate positive signals in the yeast samples. (B) Isolated RNAs mixed with urea-containing sample buffer were heated at 65°C for 15 min, followed by chilling on ice, and then spotted on the membrane. Image B is identical image shown in Fig 2B.
(TIF)

## References

[1] Cantara WA, Crain PF, Rozenski J, McCloskey JA, Harris KA, Zhang X, et al The RNA Modification Database, RNAMDB: 2011 update. Nucleic Acids Res. 2011;39(Database issue):D195–201. 10.1093/nar/gkq1028 21071406 PMC3013656

[2] Machnicka MA, Milanowska K, Osman Oglou O, Purta E, Kurkowska M, Olchowik A, et al MODOMICS: a database of RNA modification pathways—2013 update. Nucleic Acids Res. 2013;41(Database issue):D262–7. 10.1093/nar/gks1007 23118484 PMC3531130

[3] Klungland A, Dahl JA. Dynamic RNA modifications in disease. Curr Opin Genet Dev. 2014;26:47–52. 10.1016/j.gde.2014.05.006 . 25005745

[4] Kirchner S, Ignatova Z. Emerging roles of tRNA in adaptive translation, signalling dynamics and disease. Nat Rev Genet. 2015;16(2):98–112. 10.1038/nrg3861 . 25534324

[5] Ge J, Yu YT. RNA pseudouridylation: new insights into an old modification. Trends Biochem Sci. 2013;38(4):210–8. 10.1016/j.tibs.2013.01.002 23391857 PMC3608706

[6] Motorin Y, Helm M. tRNA stabilization by modified nucleotides. Biochemistry. 2010;49(24):4934–44. 10.1021/bi100408z . 20459084

[7] Mishima E, Inoue C, Saigusa D, Inoue R, Ito K, Suzuki Y, et al Conformational change in transfer RNA is an early indicator of acute cellular damage. Journal of the American Society of Nephrology: JASN. 2014;25(10):2316–26. 10.1681/ASN.2013091001 24833129 PMC4178440

[8] Kirino Y, Yasukawa T, Ohta S, Akira S, Ishihara K, Watanabe K, et al Codon-specific translational defect caused by a wobble modification deficiency in mutant tRNA from a human mitochondrial disease. Proc Natl Acad Sci U S A. 2004;101(42):15070–5. 10.1073/pnas.0405173101 15477592 PMC524061

[9] Zheng G, Dahl JA, Niu Y, Fedorcsak P, Huang CM, Li CJ, et al ALKBH5 is a mammalian RNA demethylase that impacts RNA metabolism and mouse fertility. Mol Cell. 2013;49(1):18–29. 10.1016/j.molcel.2012.10.015 23177736 PMC3646334

[10] Jia G, Fu Y, Zhao X, Dai Q, Zheng G, Yang Y, et al N6-methyladenosine in nuclear RNA is a major substrate of the obesity-associated FTO. Nature chemical biology. 2011;7(12):885–7. 10.1038/nchembio.687 22002720 PMC3218240

[11] Saikia M, Fu Y, Pavon-Eternod M, He C, Pan T. Genome-wide analysis of N1-methyl-adenosine modification in human tRNAs. RNA. 2010;16(7):1317–27. 10.1261/rna.2057810 20484468 PMC2885681

[12] Iwanami Y, Brown GM. Methylated bases of ribosomal ribonucleic acid from HeLa cells. Arch Biochem Biophys. 1968;126(1):8–15. . 5671075 10.1016/0003-9861(68)90553-5

[13] Bringmann P, Luhrmann R. Antibodies specific for N6-methyladenosine react with intact snRNPs U2 and U4/U6. FEBS Lett. 1987;213(2):309–15. . 2951275 10.1016/0014-5793(87)81512-0

[14] Meyer KD, Saletore Y, Zumbo P, Elemento O, Mason CE, Jaffrey SR. Comprehensive analysis of mRNA methylation reveals enrichment in 3' UTRs and near stop codons. Cell. 2012;149(7):1635–46. 10.1016/j.cell.2012.05.003 22608085 PMC3383396

[15] Motorin Y, Lyko F, Helm M. 5-methylcytosine in RNA: detection, enzymatic formation and biological functions. Nucleic Acids Res. 2010;38(5):1415–30. 10.1093/nar/gkp1117 20007150 PMC2836557

[16] Mizugaki M, Itoh K, Yamaguchi T, Ishiwata S, Hishinuma T, Nozaki S, et al Preparation of a monoclonal antibody specific for 5-methyl-2'-deoxycytidine and its application for the detection of DNA methylation levels in human peripheral blood cells. Biol Pharm Bull. 1996;19(12):1537–40. . 8996634 10.1248/bpb.19.1537

[17] Ohtsuki T, Watanabe Y. T-armless tRNAs and elongated elongation factor Tu. IUBMB Life. 2007;59(2):68–75. 10.1080/15216540701218722 . 17454297

[18] Sakurai M, Ohtsuki T, Watanabe K. Modification at position 9 with 1-methyladenosine is crucial for structure and function of nematode mitochondrial tRNAs lacking the entire T-arm. Nucleic Acids Res. 2005;33(5):1653–61. 10.1093/nar/gki309 15781491 PMC1069008

[19] Yamasaki S, Ivanov P, Hu GF, Anderson P. Angiogenin cleaves tRNA and promotes stress-induced translational repression. The Journal of cell biology. 2009;185(1):35–42. 10.1083/jcb.200811106 19332886 PMC2700517

[20] Dominissini D, Moshitch-Moshkovitz S, Schwartz S, Salmon-Divon M, Ungar L, Osenberg S, et al Topology of the human and mouse m6A RNA methylomes revealed by m6A-seq. Nature. 2012;485(7397):201–6. 10.1038/nature11112 . 22575960

[21] Itoh K, Mizugaki M, Ishida N. Preparation of a monoclonal antibody specific for 1-methyladenosine and its application for the detection of elevated levels of 1-methyladenosine in urines from cancer patients. Japanese journal of cancer research: Gann. 1988;79(10):1130–8. . 3143701 10.1111/j.1349-7006.1988.tb01536.x PMC5917635

[22] Itoh K, Mizugaki M, Ishida N. Detection of elevated amounts of urinary pseudouridine in cancer patients by use of a monoclonal antibody. Clinica chimica acta; international journal of clinical chemistry. 1989;181(3):305–15. . 2758683 10.1016/0009-8981(89)90236-2

[23] Erlanger BF, Beiser SM. Antibodies Specific for Ribonucleosides and Ribonucleotides and Their Reaction with DNA. Proc Natl Acad Sci U S A. 1964;52:68–74. 14192660 10.1073/pnas.52.1.68 PMC300575

[24] Kikusato M, Sudo S, Toyomizu M. Methionine deficiency leads to hepatic fat accretion via impairment of fatty acid import by carnitine palmitoyltransferase I. Br Poult Sci. 2015;56(2):225–31. 10.1080/00071668.2014.996529 . 25561085

[25] Echtay KS, Esteves TC, Pakay JL, Jekabsons MB, Lambert AJ, Portero-Otin M, et al A signalling role for 4-hydroxy-2-nonenal in regulation of mitochondrial uncoupling. EMBO J. 2003;22(16):4103–10. 10.1093/emboj/cdg412 12912909 PMC175801

[26] Russell SP, Limbach PA. Evaluating the reproducibility of quantifying modified nucleosides from ribonucleic acids by LC-UV-MS. J Chromatogr B Analyt Technol Biomed Life Sci. 2013;923–924:74–82. 10.1016/j.jchromb.2013.02.010 23500350 PMC3742090

[27] Saigusa D, Suzuki N, Takahashi M, Shiba K, Tanaka S, Abe T, et al Simultaneous determination of guanidinosuccinic acid and guanidinoacetic acid in urine using high performance liquid chromatography/tandem mass spectrometry. Anal Chim Acta. 2010;677(2):169–75. 10.1016/j.aca.2010.08.005 . 20837184

[28] Mishima E, Fukuda S, Shima H, Hirayama A, Akiyama Y, Takeuchi Y, et al Alteration of the Intestinal Environment by Lubiprostone Is Associated with Amelioration of Adenine-Induced CKD. Journal of the American Society of Nephrology: JASN. 2015;26(8):1787–94. 10.1681/ASN.2014060530 25525179 PMC4520171

