# Supplementary figures and images for: Immuno-Northern Blotting: Detection of RNA Modifications by Using Antibodies against Modified Nucleosides
Source: PLoS One. 2015 Nov 25;10(11):e0143756. doi: 10.1371/journal.pone.0143756 (PMC4659547; doi:10.1371/journal.pone.0143756)

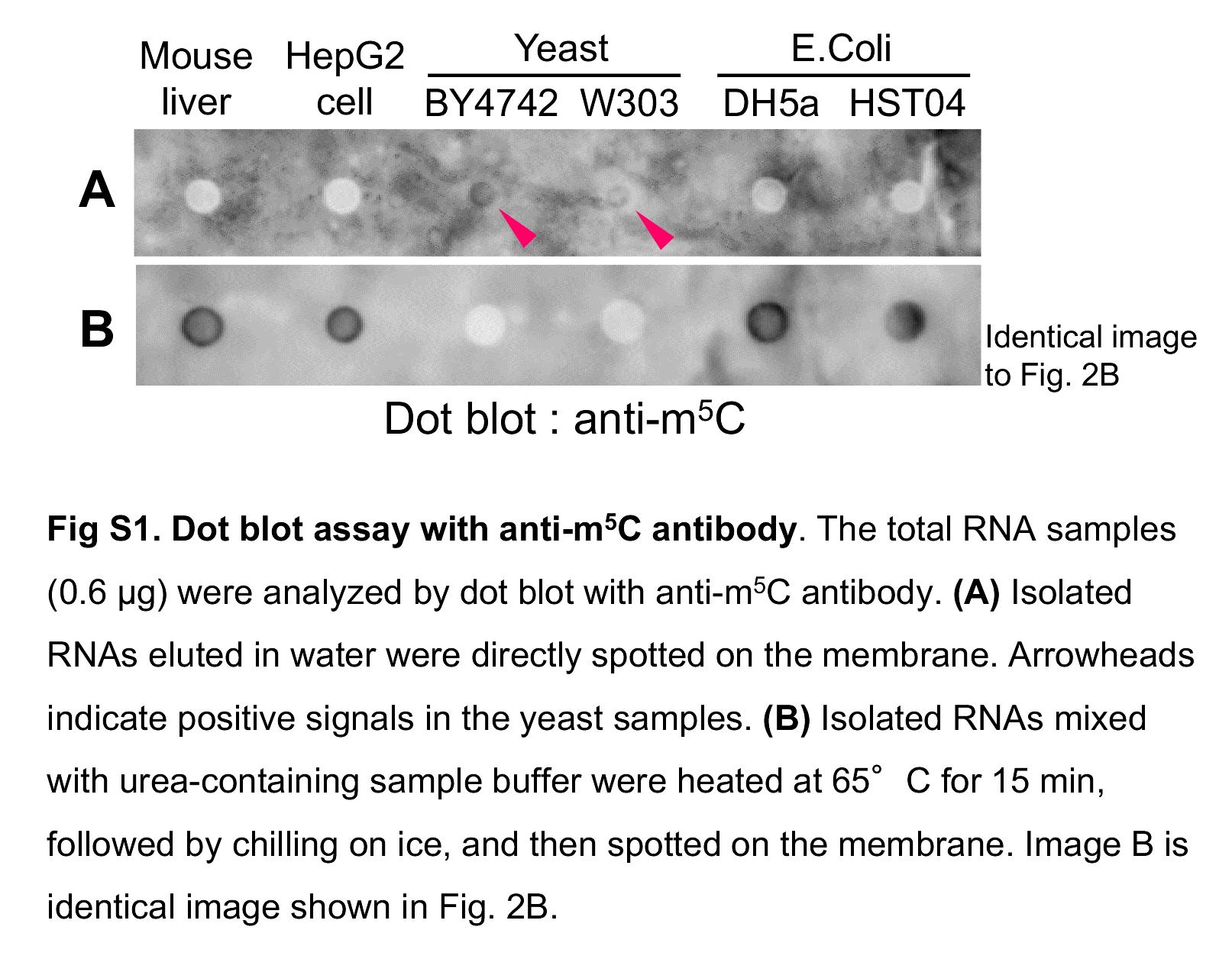

Supplement: S1 Fig — The total RNA samples (0.6 μg) were analyzed by dot blot with anti-m5C antibody. (A) Isolated RNAs eluted in water were directly spotted on the membrane. Arrowheads indicate positive signals in the yeast samples. (B) Isolated RNAs mixed with urea-containing sample buffer were heated at 65°C for 15 min, followed by chilling on ice, and then spotted on the membrane. Image B is identical image shown in Fig 2B. (TIF) [file pone.0143756.s001.tif]
